# Cross-talk between inflammation and coagulation in severe COVID-19: Association of leptin and classical pro-inflammatory markers with coagulation disorders in a single-center observational cohort study

**DOI:** 10.1097/MD.0000000000048553

**Published:** 2026-05-01

**Authors:** Loredana Camelia Boicean, Rares Mircea Birlutiu, Victoria Birlutiu

**Affiliations:** a Department of Medical Sciences, Faculty of Medicine, “Lucian Blaga” University of Sibiu, Sibiu, Romania; b Department of Infectious Diseases, Academic Emergency Hospital Sibiu, Sibiu, Romania; c Department of Orthopaedics and Traumatology, “Carol Davila” University of Medicine and Pharmacy, Bucharest, Romania; d Department of Orthopaedics, Foisor Orthopaedics Clinical Hospital, Bucharest, Romania.

**Keywords:** classical biomarkers, coagulation, COVID-19, leptin, SARS-CoV-2 infection

## Abstract

We performed a single-center observational cohort study on patients with COVID-19. We retrospectively analyzed 54 patients with confirmed SARS-CoV-2 infection, diagnosed as critically ill. Severely ill patients have a poor prognosis and it is a matter of great interest to identify these cases for an adequate management. Current findings revealed that altered levels of some blood markers might be linked with the degree of severity and mortality of patients with COVID-19. Our study aimed to assess the relationship between inflammation and coagulation in SARS-CoV-2 infection and to find out if pro-inflammatory markers are correlated with coagulation parameters in COVID-19. Pro-inflammatory markers included leptin and classical biomarkers. This paper highlights the results obtained. We found statistically significant associations between blood levels of various biomarkers including leptin, IL-6, ferritin, neutrophil-lymphocyte ratio, C-reactive protein, fibrinogen, erythrocyte sedimentation rate and lactate dehydrogenase and the presence of coagulopathy, as indicated by the Pearson Chi-Square and Likelihood Ratio tests. The relationships are not linear, as indicated by the nonsignificant Linear-by-Linear Association test. The correlations between some biomarkers such as leptin, IL-6, ferritin, neutrophil-lymphocyte ratio, C-reactive protein, fibrinogen, erythrocyte sedimentation rate and lactate dehydrogenase and coagulopathy are weak and not statistically significant and the correlation between IL-6 levels and coagulopathy is very weak and negative and not statistically significant. Different crosstabulations between serum leptin levels and D-dimers, Prothrombin time, Prothrombin activity, International-normalized-ratio and activated partial thromboplastin time have been performed including the analysis of a potentional correlation. There are statistically significant associations between serum leptin levels and coagulation parameters, including D-dimers, prothrombin time, prothrombin activity and activated partial thromboplastin time, as indicated by the Pearson Chi-Square test. There is also a statistically significant association between serum leptin levels and international-normalized ratio (INR) as indicated by the Pearson Chi-Square test. The Linear-by-Linear Association test indicates a significant linear relationship between Leptin levels and international-normalized ratio. Pearson R shows a moderate positive correlation, which is statistically significant.

## 1. Introduction

Coronavirus disease 2019 (COVID-19) is an infectious disease with origin in Wuhan, China, caused by severe acute respiratory syndrome coronavirus 2 (SARS-CoV-2), with first confirmed cases in December 2019. It is a complex disease with a wide spectrum of clinical presentations, ranging from asymptomatic infection to multi-organ failure and death. The virus spread rapidly to other countries across the world, which led World Health Organization to declare a Public Health Emergency of International Concern at the beginning of the year 2020 and to characterize the outbreak as a pandemic on March 2020. COVID-19 pandemic led to a collapse of all healthcare systems, with more than 110 million confirmed cases and more than 7 million deaths worldwide.^[1–3]^

Severely ill patients have a poor prognosis and it is a matter of great interest to identify these cases for an adequate management. Current findings revealed that altered levels of some blood markers might be linked with the degree of severity and mortality of patients with COVID-19. Our study aimed to assess the relationship between inflammation and coagulation in SARS-CoV-2 infection and to find out if pro-inflammatory markers are correlated with coagulation parameters in COVID-19.

This paper highlights the results obtained after enrolling a number of 54 patients in the study. All patients were admitted to the Infectious Diseases Clinic of the Academic Emergency Hospital Sibiu with confirmed SARS-CoV-2 infection, diagnosed as critically ill.

While the interaction between inflammation and coagulation in COVID-19 has been extensively investigated, the contribution of adipokines, particularly leptin, to coagulation abnormalities in critically ill patients remains insufficiently characterized. The present study explores leptin within the framework of COVID-19-associated thrombo-inflammation, aiming to evaluate statistical associations rather than causal biological relationships.

## 2. Materials and methods

### 2.1. Study design

We performed a single-center observational cohort study on patients with COVID-19. The study was conducted at the Infectious Diseases Clinic of Academic Emergency Hospital Sibiu, Romania, from May 1st, 2020 until November 30, 2020. We retrospectively analyzed 54 patients aged over 18 years, from which 24 were female patients and 30 were male patients, all with confirmed SARS-CoV-2 infection, diagnosed as critically ill. Our objective was to assess the correlations between pro-inflammatory markers and coagulation parameters in SARS-CoV-2 infection. Pro-inflammatory markers included leptin and classical biomarkers.

### 2.2. Sample size

We screened for eligibility hospitalized patients with SARS-CoV-2 confirmed infection who were admitted to the Infectious Diseases Clinic of Academic Emergency Hospital Sibiu, Romania, during the first 2 SARS-CoV-2 infection waves.

A total of 132 patients were screened for eligibility based on the inclusion and exclusion criteria listed below. Out of the total number of screened patients, 54 of them were found to be eligible and enrolled in the study.

### 2.3. Inclusion and exclusion criteria

The study included patients with clinical and epidemiological characteristics of COVID-19, as well as laboratory and radiological examination results suggestive of the disease.

The following inclusion criteria were considered for enrollment in the study: patients aged over 18 years, who met the diagnostic criteria for SARS-CoV-2 infection: clinical manifestations (fever, dyspnea, cough, sore throat, fatigue, myalgia, lack of appetite, nausea, headache, diarrhea, recent onset of anosmia or ageusia in the absence of any other identified cause), radiological findings of consolidation and/or ground-glass opacities on chest X-ray or computed tomography, real-time reverse transcription-polymerase chain reaction (RT-PCR) of nasal and oropharyngeal swab positive for SARS-CoV-2.

We screened patients for severe disease criteria: severe pneumonia as defined by the Chinese Centers for Disease Control and Prevention: dyspnea, tachypnea (respiratory rate >30 breaths/min), SpO2 <93%, PaO_2_/FiO_2_ <300 mm Hg, increase in the size of lung lesions by over 50% in 24 to 48 hours. Patients who presented respiratory distress, septic shock, and/or multiple organ dysfunction syndrome were diagnosed as critically ill.^[4,5]^

Patients who tested negative for SARS-CoV-2 infection, patients with bacterial, interstitial or eosinophilic pneumonia, patients on immunosuppressive drugs, including long-term use of glucocorticoids and patients who did not sign the informed consent form to participate in the study were excluded.

In addition, patients with previously known coagulation disorders were excluded from the study. Patients on anticoagulant therapy were also excluded, with the exception of patients who were on enoxaparin.

### 2.4. Data collection

Patient demographic characteristics were recorded. We recorded the gender and age, clinical manifestations of COVID-19, comorbidities and medication history, including immunosuppressive therapy. Regarding comorbidities we mention heart diseases, diabetes mellitus, lung diseases and obesity.

Informed consent was taken from the enrolled patients and their blood sample was drawn within 24 hours of admission. Complete blood count, neutrophil/lymphocyte ratio (NLR), C-reactive protein (CRP), serum fibrinogen, erythrocyte sedimentation rate, glycemia, creatinine, urea, serum electrolytes, transaminases, lactate dehydrogenase (LDH), interleukin-6 (IL-6), ferritin, serum leptin, D-dimer, fibrin monomers, prothrombin time, prothrombin activity (PA), international-normalized ratio (INR) and activated partial thromboplastin time (APTT), were assessed. Procalcitonin and sputum or blood culture were indicated in selected cases. All patients were sent for a computed tomography scan of the chest.

### 2.5. Ethical approval

The study was reviewed and approved by the hospital research ethics committee. The study was conducted according to the guidelines of the Declaration of Helsinki.

### 2.6. Data analysis

Statistical analyses were performed using the IBM SPSS Statistics version 29 software (Chicago). The analysis of continuous and categorical variables expressed as counts and percentage was performed using univariate descriptive statistics. We performed the Mann–Whitney test to compare continuous variables as distributions if they were skewed. In contrast, we used the chi-square, Fisher exact, or Cramer V tests for categorical variables. To assess the normal distribution of the data, we utilized both Kolmogorov–Smirnov and Shapiro–Wilk tests. A value of *P* .05 or less was considered significant.

Given the exploratory nature of the study and the relatively small sample size, the statistical analyses were primarily focused on descriptive and bivariate association testing. Multivariable regression modeling was not performed to avoid model overfitting and unstable estimates, and therefore the analyses were intended to identify potential associations rather than independent predictors.

## 3. Results

### 3.1. Study population

Of the total number of hospitalized patients in our clinic during the study period, 54 patients met the inclusion criteria in our study, and all included cases had a complete electronic medical record. Beside our main aim of this analysis, we also aimed to retrospectively evaluate 40 patients that presented during the hospitalization period coagulation disorders (CD group) versus 14 patients without coagulation disorders during the hospitalization period (non-CD group).

As per our previously published results from this study,^[6]^ the study population had a slight male predominance, with males making up to 55.6% (n = 30) of the participants. The ages of the 54 patients ranged between a minimum of 28 and a maximum of 80 years, with a mean of 50.58 years and a standard deviation of ± 13.37 years. The mean age of the 40 patients with coagulation disorders during the hospitalization period was 51.50 years and a standard deviation of 13.63 years, versus the 14 patients without coagulation disorders during hospitalization period a mean age of 49.00 years and a standard deviation of 12.93 years.

A high prevalence of obesity (BMI >30 kg/m^2^) was observed among patients enrolled in our study, accounting for 63% of the participants.

Of the 40 patients with coagulopathy, 36 (90%) had a favorable outcome, while 4 (10%) experienced death. All 14 patients without coagulopathy had a favorable outcome. A crosstabulation between the presence of coagulopathy and the outcome was performed. The Pearson Chi-Square value of 1.512 (df = 1, *P* = .219) indicates no significant association between coagulopathy and patient outcomes (favorable or death) in our cohort of patients. The results underscore the need for larger, more detailed studies to fully understand the factors influencing patient outcomes in the presence of coagulopathy. Also, a possible relationship between coagulopathy, serum leptin levels, and patient outcomes (favorable or death) was explored, and a statistically significant association between serum leptin levels and patient outcomes in the presence of coagulopathy, as indicated by the Pearson Chi-Square test (value 40.00, df = 17, *P* = .001) was found.

The baseline characteristics of the study participants are summarized in Table 1.

**Table 1:** Baseline characteristics of the enrolled patients.

Variables		N = 54	CD group of patients (n = 40)	Non-CD group of patients (n = 14)	*P* Pearson Chi-Square
Age (years)–mean, std. dev.		59.85, ±13.37	51.50, ±13.63	49.0, ±12.93	*.00*
Gender—male (n = %)		30, 55.6%	24, 65%	6, 42.85%	*.26*
BMI > 30 kg/m^2^ (n=)		34	26	8	*.60*
Outcome	Favorable	50	36	14	*.29*
	Death	4	4	0	
Pneumonia severity score - mean, std. dev.		15.37, ±5.32	15.45, ±5.00	15.14, ±6.359	*.39*

The italic values indicate statistically significant results (*P* < .05).

BMI = body mass index, CD group = patients with coagulation disorders, non-CD group = patients without coagulation disorders.

### 3.2. Laboratory differences between groups

A detailed insight into the distribution and variability of the main studied laboratory parameters and imaging diagnostic scores within the studied population was performed and summarized in Table 2. This analysis provides a comprehensive comparison of various laboratory parameters between patients with coagulopathy (CD group) and those without coagulopathy (Non-CD group). The significant differences in several parameters suggest potential biomarkers for monitoring these 2 groups. Specifically, leptin, IL-6, ferritin, NLR, CRP, fibrinogen, ESR, LDH, D-dimers, PA, and INR showed significant differences between the groups, indicating their potential relevance in clinical assessments and management of coagulopathy and also in the potential outcome of these patients. Prothrombin Time did not show a significant difference, indicating it might not be a distinguishing factor between the groups.

**Table 2:** Laboratory parameters within the studied population.

		Minimum	Maximum	Mean	Std. deviation	*P* Pearson Chi-Square
Leptin (adjusted for gender and body mass index)	N = 54	4.20	82.00	34.33	23.79	–
	CD group	4.20	82.00	24.84	36.68	<.00
	Non-CD group	7.20	68.00	27.62	19.79	
Interleukin-6 (0–7 pg/mL)	N = 54	38.00	8507.00	1024.35	1765.12	–
	CD group	38.00	8507.00	1021.97	1976.06	.00
	Non-CD group	44.00	2542.00	1031.14	993.80	
Ferritin (10–291 ng/mL)	N = 54	68.00	1798.50	754.87	495.00	–
	CD group	68.00	1798.50	800.43	479.04	.00
	Non-CD group	132.30	1623.60	624.70	534.68	
Neutrophil-lymphocyte ratio	N = 54	2.2970	72.5830	17.314	17.00	–
	CD group	2.29	72.58	18.92	19.09	.00
	Non-CD group	2.78	25.17	12.71	7.32	
C-reactive protein (0–5 mg/L)	N = 54	12.52	334.70	121.82	78.03	–
	CD group	12.52	334.70	117.65	81.05	.00
	Non-CD group	14.25	258.70	133.73	70.06	
Fibrinogen (180–380 mg/dL)	N = 54	225.10	786.00	553.65	155.20	–
	CD group	225.10	786.00	561.84	145.01	.00
	Non-CD group	280.10	786.00	530.27	185.55	
Erythrocyte sedimentation rate (0–15 mm/h)	N = 54	3	105	41.44	26.95	–
	CD group	7	104	43.45	24.26	.00
	Non-CD group	3	105	35.71	33.89	
Lactate dehydrogenase (135–214 U/L)	N = 54	183	761	381.89	127.25	–
	CD group	225	761	386.60	130.67	.00
	Non-CD group	18.	496	368.43	120.56	
D-dimers (45–499 ng/mL)	N = 54	189.64	7385.43	1038.01	1391.02	–
	CD group	199.53	7385.43	1300.89	1533.21	.00
	Non-CD group	189.64	539.87	286.94	127.63	
Prothrombin time (9.8–12.2 s)	N = 54	10.3	17.3	12.22	1.35	–
	CD group	10.3	17.3	12.31	1.50	.12
	Non-CD group	10.8	13.2	11.95	.78	
Prothrombin activity (82%–126%)	N = 54	47.7	122.1	90.82	15.44	–
	CD group	47.7	122.1	89.69	17.11	.02
	Non-CD group	83.3	109.9	94.04	8.83	
International-normalized-ratio (0.86–1.1)	N = 54	.90	1.56	1.093	.15	–
	CD group	.90	1.56	1.10	.17	.02
	Non-CD group	.95	1.25	1.06	.09	
Activated partial thromboplastin time (24.8–37.5 s)	N = 54	17.2	45.1	30.71	6.50	–
	CD group	17.2	45.1	30.42	7.09	<.00
	Non-CD group	25.6	37.3	31.52	4.56	

CD group = patients with coagulation disorders, non-CD group = patients without coagulation disorders.

### 3.3. Inflammatory biomarkers and coagulopathy

Multiple inflammatory biomarkers, including IL-6, ferritin, NLR, CRP, fibrinogen, ESR, and LDH, demonstrated statistically significant categorical associations with the presence of coagulopathy, as indicated by the Pearson Chi-Square and Likelihood Ratio tests.

The relationship is not linear, as indicated by the nonsignificant Linear-by-Linear Association test. The correlation between them and coagulopathy is weak and negative and not statistically significant.

Results were consistent across categorical association tests; detailed statistics are presented in Table 2.

### 3.4. Leptin and coagulation parameters

There is a statistically significant association between Leptin levels and the presence of coagulopathy, as indicated by the Pearson Chi-Square (χ2(21) = 48.793, *P* <.001) and likelihood ratio tests (χ2(21) = 56.261, *P* <.001). The relationship is not linear, as indicated by the nonsignificant Linear-by-Linear Association test (χ2(1) = 1.502, *P* = .220). The correlation between Leptin levels and coagulopathy is weak (*R* = 0.168, *ρ* = 0.168), and not statistically significant (*P* = .224).

We found is a statistically significant association between serum leptin levels and several coagulation parameters (D-dimers, prothrombin time, PA, and APTT) as indicated by the Pearson Chi-Square test. However, this is not supported by the Likelihood Ratio test and the Linear-by-Linear Association analysis indicates that there is no significant linear relationship between Leptin levels and these coagulation parameters. Both Pearson R and Spearman correlation show a very weak correlation between serum leptin levels and D-dimers, prothrombin time, PA, or APTT, and these correlations are not statistically significant.

In contrast, leptin levels showed a significant linear association with INR, as indicated by the Pearson Chi-Square test (*χ*2(378) = 792.000, *P* <.001). The Linear-by-Linear Association test (*χ*2(1) = 9.326, *P* = .002) indicates a significant linear relationship between Leptin levels and INR, and Pearson R shows a moderate positive correlation (*R* = 0.419, *P* = .002), which is statistically significant, indicating a moderate positive relationship between leptin and coagulation dysfunction.

## 4. Discussion

Given the retrospective observational design and limited sample size, the present study evaluates statistical associations between inflammatory biomarkers and coagulation parameters and does not allow inference of biological causality.

### 4.1. The inflammatory response in SARS-CoV-2 infection

One of the major challenges in COVID-19 is the high inflammatory state it provokes. It induces a disproportionate inflammatory response in the host that may cause life threatening complications.^[1]^ Coronaviruses usually infect the upper respiratory tract, causing minor symptoms. SARS-CoV-2 can also replicate in the lower respiratory tract and cause pneumonia. It can lead to a hyper-inflammatory state, known as the “cytokine storm” and thrombotic events in different organs.^[1,7–9]^

The pathophysiological mechanisms of SARS-CoV-2 infection involve direct cytopathic effect induced by the viral contagion and nonregularized inflammatory replication of the host cells. The first phase is marked by death and injury of virus-infected cells as part of the virus replicative cycle. Viral replication in airways causes high levels of virus-linked pyroptosis, which is a highly inflammatory form of programmed cell death.^[1,10–12]^ The segregation of the interleukin-1β (IL-1β), a cytokine released during pyroptosis, the identification of pathogen-associated molecular patterns and damage-associated molecular patterns by alveolar epithelial cells and macrophages, induces local inflammation characterized by increased secretion of pro-inflammatorycytokines and chemokines, monocyte chemoattractant protein-1 (MCP-1), interferon gamma-inducible protein-10 (IP-10), interferon gamma (IFN-γ) and IL-6. Cytokines and chemokines induce recruitment of monocytes and T lymphocytes to the lungs and subsequent lymphopenia and increased NLR, which are seen in 80% of cases SARS-CoV-2 infection.^[1,13,14]^

Most patients recover from the disease, but there are cases when there is a defective immune response with an uncontrolled cytokine secretion and widespread pulmonary inflammation, leading to an abnormal inflammatory replication of the host cells.^[1]^

Patients can have high blood plasma levels of Interleukin-2 (IL-2), Interleukin-7 (IL-7), Interleukin-7 (IL-10), granulocyte colony-stimulating factor (G-CSF), Interferon gamma-inducible protein-10 (IP-10), MCP-1, macrophage inflammatory protein 1α (MIP1α) and tumor necrosis factor (TNF). IL-6 levels continue rising as the disease progresses and are higher in non-survivors versus survivors.^[1,13,15–17]^

The direct injury by the virus together with an uncontrolled inflammatory response, via excessive secretion of proteases and reactive oxygen species, can induce damage to the lungs, with desquamation of alveolar cells, hyaline membrane formation and pulmonary edema, leading to gas exchange deficiency, breathing difficulty and low blood oxygen levels, leaving the lungs susceptible to other infections. The unrestrained inflammatory activity can induce systemic effects. High levels of cytokines can cause septic shock and multi-organ failure.^[1,18,19]^

### 4.2. Activation of coagulation cascade in SARS-CoV-2 infection

SARS-CoV-2 infection induces an exacerbated activation of cytokines and increases the frequency of thrombotic events in patients. Coagulation disorders are frequent in SARS-CoV-2 infection.^[20]^ Coagulation alterations are primarily mediated by platelet activation and related to virus-mediated endothelial inflammation.^[21]^

IL-6 is responsible for one of the main mechanisms by which inflammation activates coagulation by inducing tissue factor (TF) expression.^[22]^ The expression of TF on monocytes contributes to the activation of the coagulation. Procoagulant phospholipids, including phosphatidylserine, and scramblase have important role in the activation of coagulation cascade. The SARS-CoV-2 genome encodes for a cation channel that stimulates the action of scramblase.^[23,24]^

Coagulation cascade is also activated by inflammation through other pathways. Cytokines, especially IL-6, induce the production of coagulation factors, which explains hyperfibrinogenemia in COVID-19. Polyphosphates derived from microorganisms activate platelets and factor XII. Neutrophil extravascular traps are released by neutrophils in SARS-CoV-2 infection, more than in other lung infections, and may contribute to thrombosis via activation of the intrinsic coagulation pathway (factor XII). Activated complement components induce cell activation.^[24–27]^

In the acute inflammatory state, there is a transient increase in tissue plasminogen activator (tPA), followed by the release of inhibitors of tPA, such as plasminogen activator inhibitor 1 (PAI-1) and thrombin-activatable fibrinolysis inhibitor. This leads to a transition from hyperfibrinolysis to hypofibrinolysis, explaining the impaired fibrinolysis in COVID-19.^[24,26]^

### 4.3. Thromboembolic events in SARS-CoV-2 infection

The incidence of thrombotic events (deep vein thrombosis, pulmonary thromboembolism, acute myocardial infarction, ischemic stroke and arterial embolism) in COVID-19 varies in the studies published in literature, probably because of the different diagnostic methods and inclusion criteria used in those studies. A recent study reported an incidence of up to 27% of thromboembolism in patients hospitalized with SARS-CoV-2 infection.^[20,28]^ Another study reported an incidence of 5.2% thrombotic events, such as venous thromboembolism (2.7%), acute ischemic stroke (1.2%) and acute myocardial infarction (1.2%).^[20,29]^ There is another study that revealed an increase in IL-1β and IL-2 blood levels in patients with SARS-CoV-2 infection who presented thrombotic events. Because of the direct tissue damage by the virus, immune cells are recruited and pro-inflammatory cytokines are generated and they act as mediators of coagulation activation.^[20,22]^

Several cytokines have been studied in SARS-CoV-2 infection. A recent study reported that there were higher serum levels of IL-1β, IL-10 and IL-2 in the group of patients with confirmed thrombotic events than in the one without thrombotic events (*P* <.05). There were also significantly higher levels of serum IL-6 and TNF- α in patients with a thrombotic event, but without statistical significance. Serum levels of IL-6 in patients with COVID-19 are positively correlated with the severity of the disease.^[20]^ Acute infections can alter hemodynamics and the coagulation systems, in such a way as to precipitate thrombotic events. IL-6 induces TF expression, being responsible for one of the main mechanisms by which inflammation activates coagulation.^[20,22]^

### 4.4. The cross-talk between inflammation and coagulation in COVID-19

Inflammation and coagulation are host-defence systems that interact with each other. Inflammation leads to activation of coagulation and coagulation modulates the inflammatory activity. Inflammation and severe infection lead to hemostatic abnormalities, ranging from minor laboratory changes to severe disseminated intravascular coagulation (DIC). Systemic inflammation leads to activation of the coagulation system, as a consequence of downregulation of physiological anticoagulant mechanisms and inhibition of fibrinolysis. Activation of coagulation may considerably affect the inflammatory process by direct and indirect mechanisms.^[30,31]^ The protein C system and the antithrombin system, the main endothelial-bound anticoagulant mechanisms, are shut off by pro-inflammatory cytokines.^[32]^

Inflammation initiates clotting, decreases the activity of anticoagulant mechanisms and alters functions of the fibrinolytic system. Pro-inflammatory cytokines and other mediators activate the coagulation system and are capable of downregulating physiologic anticoagulant pathways. Downregulation of anticoagulant pathways promotes thrombosis and amplifies the inflammatory response.^[31,33]^ Activation of coagulation and fibrin deposition as a result of inflammation have an important role in the host defense against infectious agents to limit the inflammatory response to a specific area. When the inflammation-coagulation interactions overwhelm the natural defence systems, disastrous events occur, such as severe sepsis.^[33,34]^

Critically ill patients often have activation of both inflammatory and coagulation systems. Inflammation and coagulation are an essential part in the pathogenesis of vascular disease. The interactions between inflammation and coagulation may have significant consequences for the pathogenesis of microvascular failure and subsequent multiple organ failure, as a result of severe infection and systemic inflammatory response.^[30,32]^

Severe cases of COVID-19 present elevated fibrinogen levels, intra- and extravascular fibrin deposition and fibrin degradation. High levels of fibrinogen and the fibrin-degradation product D-dimer predict an unfavorable outcome in COVID-19, due to pulmonary embolism and DIC.^[35,36]^

We found a study that evaluates the impact of SARS-CoV-2 infection in the coagulation cascade in vitro.^[37]^ A high expression of genes for fibrinogen in the mononuclear cells was reported. Current findings show that patients with SARS-CoV-2 infection present hypercoagulability and arterial and venous thrombosis, complications that seem to be related to serum leptin levels.^[37,38]^ Initial reports from Europe revealed a high incidence of thromboembolic events, mostly pulmonary embolism, among hospitalized patients with COVID-19, even in the presence of anticoagulant prevention.^[24,39–42]^ High D-dimer levels are a consistent finding in severe cases of COVID-19. High baseline and peak D-dimer levels were found to be linked to disease severity, thromboembolic events, transfer to intensive care units and overall mortality.^[39,43]^

Severely ill COVID-19 patients present a hypercoagulable state and elevated D-dimer levels are in response to this prothrombotic status.^[44]^ Multiple studies have identified a correlation between increased D-dimer levels and an increased risk of mortality in SARS-CoV-2 infection.^[45–47]^

Few authors published results including laboratory tests related to coagulation in COVID-19. A recent study revealed that PA was lower in the group with thrombotic events during SARS-CoV-2 infection. The difference between the groups included in the study was not statistically significant, but the biserial point correlation showed that there was a significant difference between the 2 groups and that if the samples were larger, it would probably lead to significant results. The APTT did not present significant differences between the analyzed groups. In both groups, PAT and APTT values were outside the reference range. This could be explained by to the complexity of the disease caused by SARS-CoV-2, which can induce an inflammatory process and changes in clotting factors and coagulation cascade, regardless of the presence of a thrombotic event.^[20]^

Several studies showed that D-Dimer, Prothrombin Time and INR are significant indicators of severe COVID-19 and poor prognosis.^[48–51]^

PT and aPTT levels are increased in patients with COVID-19. Prothrombin time and fibrinogen were reported to be higher in severe cases of COVID-19 than those in mild cases. Shorter APTT and longer prothrombin time have been reported.^[52,53]^

Another study showed that a baseline PT at a cutoff value ≥16.25 seconds differentiated severe COVID-19 patients from mild and moderate patients and a baseline PT at a cutoff value ≤ 15.35 seconds differentiated mild COVID-19 patients from moderate and severe patients. In conclusion, this study shows that prolonged baseline PT was observed in severe COVID-19 patients and predicted a poor prognosis. An increase from the baseline PT was also associated with poor prognosis. Therefore, PT can be used as a risk stratification tool and a prognostic marker in SARS-CoV-2 infection.^[49]^ There are other authors that also showed prothrombin time to be a predictor of death in COVID-19. The survival rate was significantly lower patients with prolonged prothrombin time compare to those with normal prothrombin time (*P* <.001).^[50]^

Another study showed that PT and INR are independently associated with in-hospital mortality in COVID-19.^[48]^ However, results are contradictory. In 1 study, prothrombin time and APTT were showed to be normal in patients diagnosed with COVID-19.^[54,55]^ Another study didn’t find any relationship between prothrombin time, APTT and disease severity.^[55]^

### 4.5. Pro-inflammatory markers in SARS-CoV-2 infection and coagulation disorders

#### 4.5.1. Leptin as a pro-inflammatory marker in COVID-19 and its correlations with coagulopathy and coagulation parameters

Several biological mechanisms may hypothetically explain the observed association between leptin levels and coagulation parameters. Leptin receptors are expressed on endothelial cells and may contribute to endothelial activation and TF expression during systemic inflammation. Experimental studies also suggest that leptin enhances platelet aggregation and promotes prothrombotic signaling pathways. Furthermore, obesity-related hyperleptinemia may amplify immunothrombotic responses through interaction with cytokine-mediated inflammation.

However, these mechanisms remain speculative in the context of the present study, as the observational design does not allow mechanistic validation.

Leptin, which is known more as an endocrine marker, has an important role in immunity as it acts as a pro-inflammatory cytokine and it may have a role in infection diseases and sepsis.^[56–65]^

Leptin deficiency and leptin resistance are studied as pathogenetic factors in infections and hyperleptinemia is an independent predictable factor for an unfavorable outcome.^[63–65]^ High levels of leptin induce a hyper-inflammatory state in alveolar macrophages in experimental studies, thus hyperleptinemia found in obese patients might be responsible for pulmonary inflammation in COVID-19. On the other hand, higher levels of inflammatory cytokines are produced in visceral adipose tissue compared to subcutaneous adipose tissue, suggesting that cytokine storm in SARS-CoV-2 infection might not have any link to obesity.^[66,67]^

Hypercoagulability and arterial and venous thrombosis found in COVID-19 are complications that appear to be related to total serum leptin levels, according to some studies.^[37,38]^ Leptin levels correlate with the thromboembolic risk, by a possible synergistic action with the SARS-CoV-2 virus in the capillary endothelium, and it is associated with renal impairment increasing the risk of a poor prognosis in advanced stages of renal impairment.^[68–73]^

In vitro studies showed that leptin has been associated with altered blood coagulation. However, in vivo studies need to be conducted in order to confirm this. It is also a matter of interest in what extent this association is influenced by total body fat.^[74]^

Serum leptin correlates with body mass index and obesity increases the thrombotic risk.^[75]^ We found a study published in literature that aimed to examine the association between serum leptin and blood coagulation while taking total body fat into account in a population-based cohort study. They performed a cross-sectional analysis with baseline measurements of 5797 participants. “In multivariable adjusted analyses a 1 μg/L higher serum leptin concentration was associated with a 0.22 IU/dL (95% CI: 0.11–0.32) higher FVIII concentration and a 0.20 IU/dL (95% CI: 0.14–0.27) higher FIX concentration (3.5 IU/dL FVIII and 3.2 IU/dL FIX per SD leptin). Serum leptin concentration was not associated with FXI, fibrinogen, platelet count, mean platelet volume and platelet distribution width in multivariable adjusted analyses.” This study revealed that serum leptin levels were associated with higher concentrations of FVIII and FIX, which could be clinically relevant.^[74]^

Experimental studies in mice demonstrated that leptin promotes both arterial and venous thrombosis. By directly binding to platelets and activating them, leptin enhances the expression of prothrombotic and anti proteins in inflammatory cells. Recruitment of vascular progenitor cells to sites of vascular injury was found to be impaired in hyperleptinemic obese humans and mice. Leptin promotes thrombus formation and resolution by multiple mechanisms including primary hemostasis and the coagulation cascade. These results could facilitate finding novel therapeutic options in patients with increased risk of thrombosis associated with obesity induced hyperleptinemia, taking into consideration issues of a cell-specific leptin resistance as well as individual differences in the responsiveness to leptin.^[68]^

Our study revealed a statistically significant association between Leptin levels and the presence of coagulopathy, as indicated by the Pearson Chi-Square (χ2(21) = 48.793, *P* < .001) and Likelihood Ratio tests (χ2(21) = 56.261, *P* <.001). The relationship is not linear, as indicated by the nonsignificant Linear-by-Linear Association test (χ2(1) = 1.502, *P* = .220). The correlation between Leptin levels and coagulopathy is weak (*R* = 0.168, *ρ* = 0.168), and not statistically significant (*P* = .224).

We didn’t find any study published in literature regarding correlations between serum leptin levels and coagulation parameters in COVID-19.

Crosstabulation analyses demonstrated statistical associations between leptin levels and several coagulation parameters (D-dimers, prothrombin time, PA, and APTT); however, these findings were not supported by correlation analyses and no significant linear relationships were identified.

In our study, serum leptin levels showed a statistically significant linear association with INR, supported by both linear-by-linear analysis and correlation testing (*R* = 0.419, *P* = .002), indicating a moderate positive relationship between leptin and coagulation dysfunction.

#### 4.5.2. Classical pro-inflammatory markers and the presence of coagulopathy in COVID-19

We didn’t find any study published in literature so far evaluating correlations between inflammatory markers and coagulation parameters in COVID-19.

High levels of IL-6 characterize severe coronavirus infections.^[76]^ IL-6 stimulates coagulation as reflected in increased thrombin-antithrombin III complexes and high levels of prothrombin activation of fragment F1 and F2. IL-6 stimulates the production of other cytokines, such as TNF-α and IL-1β, which can also influence the coagulation cascade and promote thrombus formation.^[77,78]^ The release of IL-6 in the cytokine storm in COVID-19, could induce pulmonary intravascular coagulation. However, the relationship between IL-6 and coagulopathy remains unclear. There is a study that found a correlation between elevated IL-6 and D-dimer levels with disease deterioration, but there was no correlation between elevated IL-6 levels with ferritin, D-dimer, fibrinogen and PT levels.^[79]^ Another study revealed higher levels of IL-6 in patients with a thrombotic event, but without statistical significance.^[20]^

The results in our study reveal a statistically significant association between IL-6 levels and the presence of coagulopathy, as indicated by the Pearson Chi-Square (*χ*2(25) = 48.793, *P* = .003) and Likelihood Ratio tests (*χ*2(25) = 56.261, *P* <.001). The relationship is not linear, as indicated by the nonsignificant Linear-by-Linear Association test (*χ*2(1) = 0.000, *P* = .987). The correlation between IL-6 levels and coagulopathy is very weak and negative (*r*=−0.002, *ρ*=−0.157), and not statistically significant (*P* = .987 and *P* = .256, respectively).

Ferritin is an important diagnostic marker and a predictor of COVID-19 severity. High levels of ferritin are found in patients with COVID-19.^[67]^ Ferritin is a mediator of the immune system through the activation of monocyte and macrophage immune cells and the synthesis of ferritin is affected by cytokine status.^[80]^ Serum ferritin is a marker of the acute-phase response and its secretion is regulated by pro-inflammatory cytokines. Hyperferritinemia is a result of the inflammatory response induced by SARS-CoV-2 infection.^[67,81,82]^

A study we found demonstrated that an increase in ferritin level could indirectly predict cytokine storm, respiratory distress syndrome and deterioration of clinical presentation of the patients.^[67,80]^

We didn’t find any study that evaluates ferritin and coagulation parameters in SARS-CoV-2 infection.

We found a statistically significant association between Ferritin levels and the presence of coagulopathy, as indicated by the Pearson Chi-Square (*χ*2(26) = 54.000, *P* = .001) and Likelihood Ratio tests (*χ*2(26) = 61.806, *P* <.001). The relationship is not linear, as indicated by the nonsignificant Linear-by-Linear Association test (*χ*2(1) = 1.307, *P* = .253). The correlation between Ferritin levels and coagulopathy is weak (*R* = 0.157, *ρ* = 0.141), and not statistically significant (*P* = .257 and *P* = .309, respectively).

Patients with elevated LDH levels were also more likely to develop critical illness. We found a study that evaluates the role of LDH in morbidity and mortality in SARS-CoV-2 infection. The study showed an association between increased LDH values and a poor prognosis in patients with COVID-19. In patients with high levels of LDH the risk of developing a severe disease was 6 times higher and the mortality rate was 16 times higher.^[83]^

In other studies reporting mortality in COVID-19, elevated LDH was found in more than 95% of non-survivors compared to <60% of survivors.^[13,84]^ Other studies compared LDH levels in severe versus non-severe cases of SARS-CoV-2 infection. 49.8% of the patients had high levels of LDH, 84.6% of them were patients with severe illness and 43.3% were patients with non-severe illness.^[13,84–89]^

We found a statistically significant association between lactate dehydrogenase levels and the presence of coagulopathy, as indicated by the Pearson Chi-Square (*χ*2(25) = 48.793, *P* = .003) and Likelihood Ratio tests (*χ*2(25) = 56.261, *P* <.001). The relationship is not linear, as indicated by the nonsignificant Linear-by-Linear Association test (*χ*2(1) = 0.211, *P* = .646). The correlation between LDH levels and coagulopathy is weak (*R* = 0.063, *ρ*=−0.005), and not statistically significant (*P* = .650 and *P* = .969, respectively).

Serum CRP is an essential marker in SARS-CoV-2 infection. High levels of CRP are correlated with the severity of the disease, indicate a higher likelihood of lung injury and a poor prognosis.

Data published in the literature so far indicates that patients with severe and critical illness had higher levels of CRP than patients with mild or moderate illness.^[90]^ High CRP levels in severe cases of COVID-19 could be related to variations in leptin levels, as CRP levels are associated with leptin levels.^[37]^ CRP regulates the function of blood platelets, the extrinsic blood coagulation cascade and the fibrinolytic system. CRP represents an important link between inflammation and thrombosis. The cross-talk between inflammation and thrombosis is bidirectional, because activation of platelets can regulate CRP structure and biological function. Further studies are needed to understand the prothrombotic effects of CRP. The results could have an important role in prevention of thrombosis by using inhibitors of CRP expression, such as statins and compounds that specifically target CRP.^[91]^

The results in the study we conducted reveal that there is statistically significant association between C-reactive protein levels and the presence of coagulopathy, as indicated by the Pearson Chi-Square (*χ*2(26) = 54.000, *P* = .001) and Likelihood Ratio tests (χ2(26) = 61.806*χ*2(26) = 61.806, *P* <.001). The relationship is not linear, as indicated by the nonsignificant Linear-by-Linear Association test (*χ*2(1) = 0.440, *P* = .507). The correlation between C-reactive proteins levels and coagulopathy is weak and negative (*r*=−0.091, *ρ*=−0.065), and not statistically significant (*P* = .512 and *P* = .640, respectively).

Fibrinogen, which is a biomarker and clotting factor, and fibrin-degradation products stimulate the expression of IL-6 mRNA and TNF-α. This reveals the close relationship between the immune system and the coagulation system in the pathogenesis of several diseases.^[20,92]^

Hyperfibrinogenemia is a predictor of poor prognosis in SARS-CoV-2 infection, leading to pulmonary embolism and DIC. A study showed that in mild cases of COVID-19 fibrinogen levels > 7.0 g/L were found in 5.7% of the patients and in severe cases were found in 19.1% of the patients. In critically ill patients, thrombolysis decreases fibrinogen levels and increases fibrin-degradation products and can lead to consumptive coagulopathy and severe bleeding.^[36]^

We found a statistically significant association between fibrinogen levels and the presence of coagulopathy, as indicated by the Pearson Chi-Square (*χ*2(22) = 41.850, *P* = .007) and Likelihood Ratio tests (*χ*2(22) = 48.623, *P* <.001). The relationship is not linear, as indicated by the nonsignificant Linear-by-Linear Association test (*χ*2(1) = 0.429, *P* = .512). The correlation between fibrinogen levels and coagulopathy is weak (*R* = 0.090, *ρ* = 0.060), and not statistically significant (*P* = .518 and *P* = .668, respectively).

NLR, an important marker of the inflammatory process, can also be a predictor of disease severity in SARS-CoV-2 infection. Investigating its value can lead to early identification and adequate treatment of severe COVID-19 pneumonia. Several studies have demonstrated that an increased NLR is associated with disease severity and mortality in COVID-19 patients, supporting its role as an accessible inflammatory prognostic marker.^[93–95]^

We found a statistically significant association between Neutrophil-Lymphocyte Ratio levels and the presence of coagulopathy, as indicated by the Pearson Chi-Square (*χ*2(26) = 54.000, *P* = .001) and Likelihood Ratio tests (*χ*2(26) = 61.806, *P* <.001). The relationship is not linear, as indicated by the nonsignificant Linear-by-Linear Association test (*χ*2(1) = 1.381, *P* = .240). The correlation between NLR levels and coagulopathy is weak (*R* = 0.161, *ρ* = 0.033), and not statistically significant (*P* = .244 and *P* = .815, respectively).

SARS-CoV-2 may induce changes in erythrocytes or plasma characteristics and increased levels of ESR levels may be a precursor of hepatic and renal dysfunction. High ESR levels can worsen outcomes in patients with SARS-CoV-2 infection, leading to various complications such as osteoarthritis.^[96]^

The results in our study reveal a statistically significant association between erythrocyte sedimentation rate levels and the presence of coagulopathy, as indicated by the Pearson Chi-Square (*χ*2(23) = 43.586, *P* = .006) and Likelihood Ratio tests (*χ*2(23) = 50.716, *P* <.001). The relationship is not linear, as indicated by the nonsignificant Linear-by-Linear Association test (*χ*2(1) = 0.854, *P* = .355). The correlation between ESR levels and coagulopathy is weak (*R* = 0.127, *ρ* = 0.185), and not statistically significant (*P* = .360 and *P* = .182, respectively).

#### 4.5.3. Study limitations:

This study has several limitations that should be considered when interpreting the findings. First, the relatively small sample size (n = 54) limits statistical power and increases the risk of type II error, potentially obscuring true associations. Second, the single-center design may introduce selection bias and restrict generalizability to broader patient populations. Third, the retrospective observational nature of the study is inherently susceptible to information and reporting bias.

Residual confounding related to age, body mass index, and comorbidities may have influenced observed associations. In addition, biomarker measurements were obtained at a single time point, preventing assessment of temporal relationships between inflammatory activation and coagulation changes.

Most statistical analyses were based on bivariate association testing, without multivariable regression modeling to adjust for potential confounders listed above. Consequently, the observed relationships cannot be interpreted as independent or causal associations. The generally weak correlation coefficients further support interpreting these findings as exploratory and hypothesis-generating.

Nevertheless, the homogeneous inclusion of critically ill patients and standardized laboratory assessment strengthen internal consistency. Larger prospective multicenter studies incorporating adjusted statistical models are required to validate and clarify the clinical relevance of the observed associations.

Future studies integrating mechanistic biomarkers and prospective validation cohorts are necessary to determine whether leptin represents a marker, mediator, or epiphenomenon of COVID-19-associated coagulopathy.

## 5. Conclusion

This study identified statistical associations between inflammatory biomarkers and coagulation parameters in critically ill patients with COVID-19, supporting the presence of a thrombo-inflammatory interaction in severe SARS-CoV-2 infection. However, the observed correlations were generally weak, and the findings should be interpreted as exploratory associations rather than evidence of biological causation.

Among the analyzed biomarkers, serum leptin demonstrated a positive correlation with INR, suggesting a potential link between adipokine signaling and coagulation imbalance in severe COVID-19. While experimental data support plausible mechanistic interactions between leptin, endothelial activation, and prothrombotic pathways, the present observational design does not allow mechanistic confirmation.

Given the retrospective single-center design, limited sample size, and absence of multivariable adjustment, these results should be considered hypothesis-generating. Prospective multicenter studies incorporating longitudinal biomarker assessment and adjusted statistical modeling are required to validate these associations and clarify their biological and clinical significance.

## Acknowledgments

This Project was financed by “Lucian Blaga” University of Sibiu & Hasso Plattner Foundation research grants LBUS-IRG-2019-05.

## Author contributions

**Conceptualization:** Loredana Camelia Boicean, Rares Mircea Birlutiu, Victoria Birlutiu.

**Data curation:** Loredana Camelia Boicean, Rares Mircea Birlutiu, Victoria Birlutiu.

**Formal analysis:** Loredana Camelia Boicean, Rares Mircea Birlutiu, Victoria Birlutiu.

**Funding acquisition:** Victoria Birlutiu.

**Investigation:** Loredana Camelia Boicean, Rares Mircea Birlutiu, Victoria Birlutiu.

**Methodology:** Loredana Camelia Boicean, Rares Mircea Birlutiu, Victoria Birlutiu.

**Project administration:** Loredana Camelia Boicean, Rares Mircea Birlutiu, Victoria Birlutiu.

**Resources:** Loredana Camelia Boicean, Rares Mircea Birlutiu, Victoria Birlutiu.

**Software:** Loredana Camelia Boicean, Rares Mircea Birlutiu, Victoria Birlutiu.

**Supervision:** Loredana Camelia Boicean, Rares Mircea Birlutiu, Victoria Birlutiu.

**Validation:** Loredana Camelia Boicean, Rares Mircea Birlutiu, Victoria Birlutiu.

**Visualization:** Loredana Camelia Boicean, Rares Mircea Birlutiu, Victoria Birlutiu.

**Writing – original draft:** Loredana Camelia Boicean, Rares Mircea Birlutiu, Victoria Birlutiu.

**Writing – review & editing:** Loredana Camelia Boicean, Rares Mircea Birlutiu, Victoria Birlutiu.

## Correction

This article was originally published with an incorrect academic title for Author “ Loredana Camelia Boicean.” This has now been updated online from “Loredana Camelia Boicean, MD, PhD” to “Loredana Camelia Boicean, MD.”
